# *Hansenula polymorpha* Pex37 is a peroxisomal membrane protein required for organelle fission and segregation

**DOI:** 10.1111/febs.15123

**Published:** 2019-11-26

**Authors:** Ritika Singh, Selvambigai Manivannan, Arjen M. Krikken, Rinse de Boer, Nicola Bordin, Damien P. Devos, Ida J. van der Klei

**Affiliations:** ^1^ Molecular Cell Biology Groningen Biomolecular Sciences and Biotechnology Institute University of Groningen the Netherlands; ^2^ Centro Andaluz de Biología del Desarrollo CSIC Universidad Pablo de Olavide Seville Spain; ^3^ Structural and Molecular Biology University College London UK

**Keywords:** peroxisome, Pex37, PXMP2, Sym1, Wsc, yeast

## Abstract

Here, we describe a novel peroxin, Pex37, in the yeast *Hansenula polymorpha*. *H. polymorpha* Pex37 is a peroxisomal membrane protein, which belongs to a protein family that includes, among others, the *Neurospora crassa* Woronin body protein Wsc, the human peroxisomal membrane protein PXMP2, the *Saccharomyces cerevisiae* mitochondrial inner membrane protein Sym1, and its mammalian homologue MPV17. We show that deletion of *H. polymorpha PEX37* does not appear to have a significant effect on peroxisome biogenesis or proliferation in cells grown at peroxisome‐inducing growth conditions (methanol). However, the absence of Pex37 results in a reduction in peroxisome numbers and a defect in peroxisome segregation in cells grown at peroxisome‐repressing conditions (glucose). Conversely, overproduction of Pex37 in glucose‐grown cells results in an increase in peroxisome numbers in conjunction with a decrease in their size. The increase in numbers in *PEX37*‐overexpressing cells depends on the dynamin‐related protein Dnm1. Together our data suggest that Pex37 is involved in peroxisome fission in glucose‐grown cells. Introduction of human PXMP2 in *H. polymorpha pex37* cells partially restored the peroxisomal phenotype, indicating that PXMP2 represents a functional homologue of Pex37. *H.polymorpha pex37* cells did not show aberrant growth on any of the tested carbon and nitrogen sources that are metabolized by peroxisomal enzymes, suggesting that Pex37 may not fulfill an essential function in transport of these substrates or compounds required for their metabolism across the peroxisomal membrane.

## Graphical abstract

In this study, van der Klei and colleagues describe the discovery of a novel peroxin, Pex37, in the yeast *Hansenula polymorpha*. Pex37 is a member of the PXMP2 family of proteins and is a peroxisomal membrane protein. The team determined that an absence of Pex37 resulted in a defect in peroxisome fission, as well as segregation between mother cells and buds. This phenotype occurred in cells grown in peroxisome‐repressing conditions, but not in peroxisome‐inducing conditions. Moreover, Pex37 overproduction resulted in a Dnm1‐dependent increase in peroxisome numbers, suggesting that Pex37 is involved in peroxisome fission.

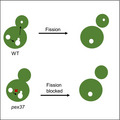

**Abbreviations**
CLSM: confocal laser scanning microscopy
EM: electron microscopy
FM: fluorescence microscopy
nm: nanometer
*PEX*: gene encoding peroxin
*Pex*: peroxin
*pex*: *PEX* deletion mutant
PMP: peroxisomal membrane protein
WB: Woronin body
WT: wild‐type

## Introduction

Peroxisomes are cell organelles that are well known for their role in a large variety of metabolic pathways. Common functions are detoxification of hydrogen peroxide and β‐oxidation of fatty acids. Examples of species‐specific functions include the biosynthesis of plasmalogens and bile acids in mammals 1, the metabolism of methanol in methylotrophic yeasts 2, and the biosynthesis of penicillin in filamentous fungi 3. Peroxisomes also can fulfill nonmetabolic functions. For instance, in filamentous ascomycetes a highly specialized peroxisome called Woronin body (WB) plugs septal pores upon hyphal wounding to prevent cytoplasmic leakage 4.

The broad range of peroxisomal metabolic pathways requires continuous metabolite exchange between the peroxisomal matrix and cytosol. So far, two pore‐forming proteins have been identified in peroxisomal membranes, namely mammalian PXMP2 5 and *Saccharomyces cerevisiae* Pex11 6. Based on *in vitro* assays and biochemical studies, both proteins were proposed to enable free diffusion of molecules with molecular masses up to 300 Da. These observations support the view that the peroxisomal membrane is permeable for small molecules, but requires specific transporters for larger ones (reviewed by 7, 8). This is further underlined by the outcome of *in vivo* polymer exclusion measurements in yeast, which pointed to a nonspecific pore in the peroxisomal membrane with a radius between 0.57 and 0.65 nm 9.

Human PXMP2 is member of a protein family, which also includes *Neurospora crassa* Woronin sorting complex (WSC), a protein of the peroxisomal and WB membrane in ascomycete fungi 10. Other members of this family include the *S. cerevisiae* mitochondrial inner membrane protein Sym1 11, its mammalian homologue MPV17 12, and *S. cerevisiae* YOR292c, a putative vacuolar protein of unknown function 13. Although members of the PXMP2 family ubiquitously occur in eukaryotes, in which they localize to various intracellular membranes, a common function for these proteins has not been established yet.

Mutations in human MPV17 result in hepatocerebral mtDNA depletion syndrome (MDDS), which is an inherited autosomal recessive disease characterized by a strongly reduced copy number of mtDNA 12. Like PXMP2, MPV17 has been suggested to function as a nonselective channel 14. Depletion of mtDNA in MDDS patients has been proposed to be caused by mitochondrial nucleotide insufficiency 15. How this relates to mutations in MPV17 is still speculative. Also, although MPV17 is an established mitochondrial inner membrane protein, a recent report indicated that it is also localized to other organelles, including peroxisomes, endosomes, and lysosomes 16. The yeast MPV17 homologue Sym1 forms a channel in the mitochondrial inner membrane and is proposed to allow passage of intermediates of the tricarboxylic acid cycle (reviewed by 17). Interestingly, deletion of *SYM1* also results in the flattening of mitochondrial cristae, suggesting a role in the maintenance of the mitochondrial ultrastructure 18.

*N. crassa* WSC has a dual function as it plays a role in WB biogenesis and segregation. WB formation depends on the peroxisomal matrix protein HEX1, which self‐assembles to produce a solid micrometer‐scale protein assembly 4, 19. This assembly associates with the matrix face of the peroxisomal membrane and subsequently buds off to form a WB. In the absence of WSC, HEX assemblies no longer associate with the peroxisomal membrane, suggesting that WSC is required to engulf HEX assemblies. WSC is also involved in cortical association of WBs as well as in proper organelle distribution 10. In addition, cortical association of WBs requires LAH, a protein that physically interacts with WSC 20. The *Aspergillus fumigatus* WSC homologue, WscA, also plays an important role in WB biogenesis, but is not required for WB segregation 21.

The above observations indicate that proteins of the PXMP2 family not only fulfill a function in solute transport, but in addition play roles in processes related to membrane shaping or organelle positioning.

In order to obtain further insights into this protein family, we studied the PXMP2 protein family in *Hansenula polymorpha,* a methylotrophic yeast that has been extensively used as a model organism for studies on peroxisome biogenesis and function. We show that one of the four PXMP2 family proteins identified in this organism localizes to peroxisomes. The absence of this protein, which we designated Pex37, resulted in a reduction in peroxisome numbers and a defect in peroxisome segregation between mother cells and buds at peroxisome‐repressing growth conditions (glucose). Upon introduction of human PXMP2 in *H. polymorpha pex37*, peroxisome numbers became normal again, indicating that this protein represents a functional homologue of Pex37.

## Results

### Identification of PXMP2 homologues in *Hansenula polymorpha*

*Saccharomyces cerevisiae* has two members of the PXMP2 family, whereas *N. crassa* and *Homo sapiens* have 5 and 4, respectively (Table 1). A search for PXMP2 family candidates in the genome of *H. polymorpha* revealed that this species has four proteins that show sequence homology with human PXMP2 and *N. crassa* WSC.

**Table 1:** Proteins of the PXMP2 family in various species.

*Saccharomyces cerevisiae*	*Hansenula polymorpha*	*Neurospora crassa*	*Homo sapiens*
Sym1 YOR292c	Hp32g403 (MN379451)	WSC (EAA33867)	PXMP2
	Hp27g68 (MN379453)	EAA34618	MPV17
	Hp24g381 (MN379452)	EAA32569	MPV17L1
	Hp32g332 (MN379454)	EAA36527	MPV17L2
		EAA33195	

**Figure 1:**
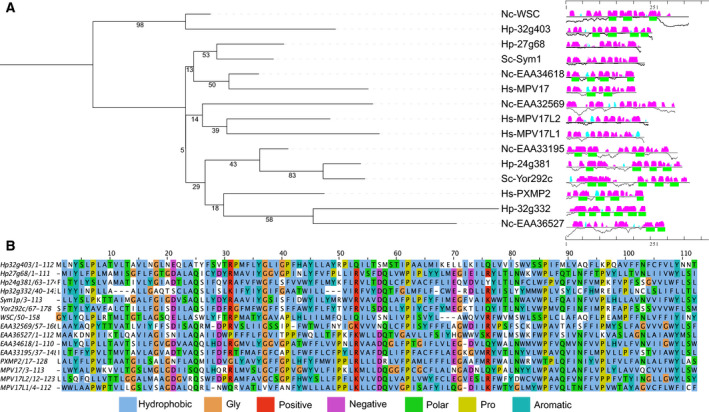
Proteins of the PXMP2 family. (A) Protein phylogeny and secondary structure features of PXMP2‐related proteins obtained with Foundation 45. Nc—*Neurospora crassa*; Sc—*Saccharomyces cerevisiae*; Hs—*Homo sapiens*; Hp— *Hansenula polymorpha*. Phylogenetic tree (left): Numbers represent the bootstraps values, while branch length represents the amino acidic substitution rates. Sequence feature representation (right): The black horizontal lines represent the protein's sequence. The predicted β‐strands and α‐helices are depicted by bars above each line in cyan and magenta, with the height of the bars representing the confidence of the prediction. Transmembrane helix predictions are depicted as green boxes underneath the secondary structure prediction. (B) Representation of a conserved portion in the sequence alignment of PXMP2 family proteins. Manually curated alignment obtained by ClustalOmega 39. Residues are colored according to their biochemical character.

In a phylogenetic tree (Fig. 1A), these proteins cluster in two major groups, one containing *N. crassa* WSC and *H. polymorpha* Hp32g403 and the other containing the rest of the proteins, including human PXMP2. An alignment of the *H. polymorpha*,* S. cerevisiae*,* N. crassa*, and human orthologs revealed four conserved regions. Hydropathy analysis of the alignment suggests that each of these conserved regions contains a hydrophobic motif that might constitute a membrane spanning domain, in agreement with transmembrane helix predictions. A short consensus sequence of 112 amino acids could be identified between the proteins (Fig. 1B).

### Hp32g403 localizes to peroxisomes

To determine the localization of the four *H. polymorpha* PXMP2 family members, we constructed strains producing C‐terminal GFP fusions, all under control of their endogenous promoter, together with the peroxisomal matrix marker DsRed‐SKL.

**Figure 2:**
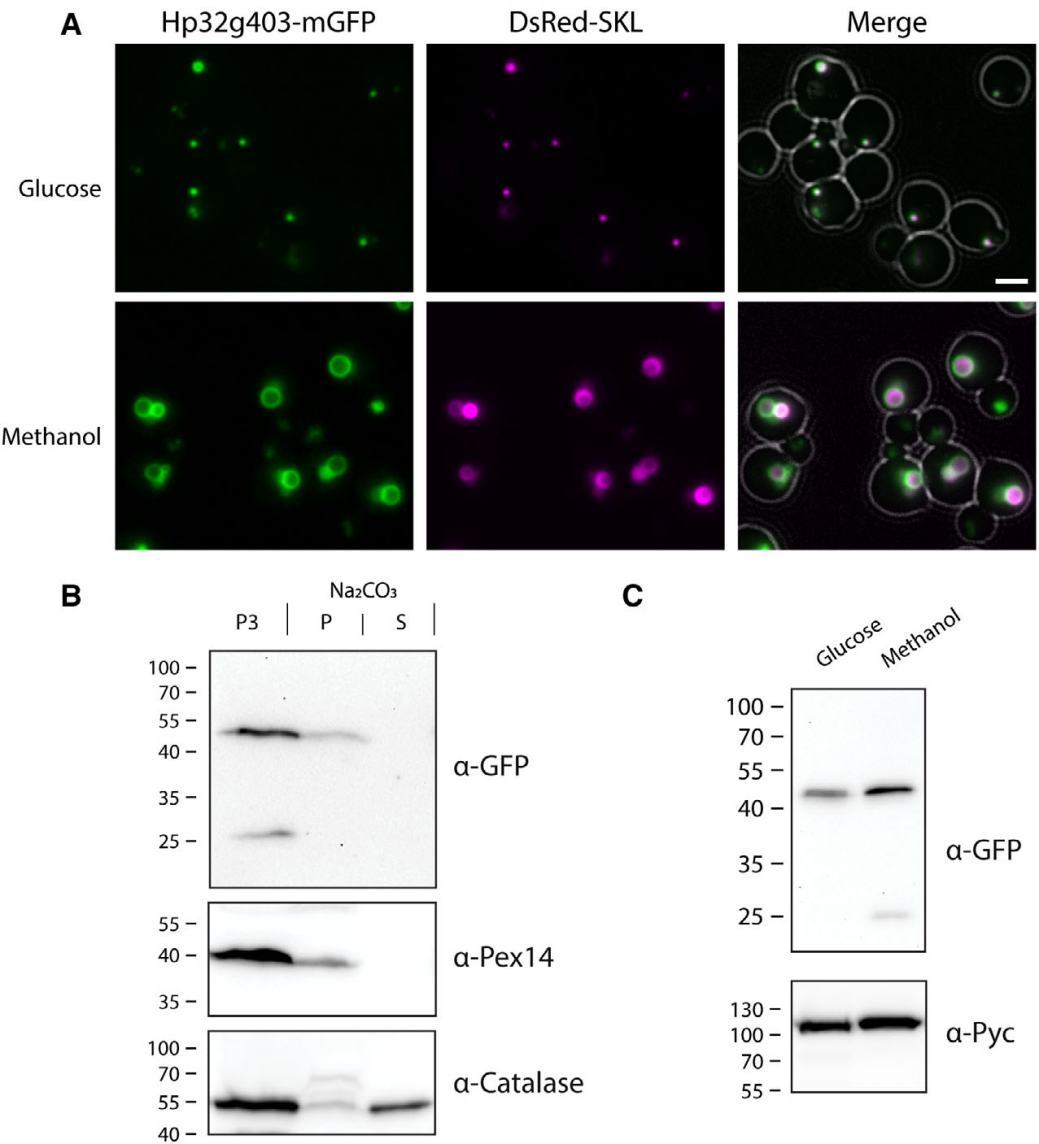
Hp32g403‐GFP localizes to peroxisomes. (A) FM images of *H. polymorpha* cells producing Hp32g403‐GFP together with DsRed‐SKL. Cells were grown to the mid‐exponential growth phase on glucose or grown for 8 h on methanol medium. In the merged image, the cell contours are indicated in white. The scale bar represents 2 μm. Representative images of two independent experiments are shown. (B) Western blot analysis of a carbonate extraction experiment using an organellar pellet (P3) of methanol‐grown WT cells producing Hp32g403‐GFP. Equal portions of the P3, pellet (P), and supernatant (S) were loaded per lane. Blots were decorated with anti‐GFP antibodies. The PMP Pex14 and matrix protein catalase were used as controls. A representative western blot of two independent experiments is shown. (C) Western blot of total cell extracts of glucose and methanol‐grown cells producing Hp32g403‐GFP. Pyruvate carboxylase (Pyc) was used as a loading control. A representative western blot of two independent experiments is shown.

Fluorescence microscopy (FM) analysis of glucose‐grown cells revealed that Hp32g403‐GFP accumulated in spots, which represent small peroxisomes based on the colocalization with DsRed‐SKL (Fig. 2). In methanol‐grown cells, multiple larger green fluorescent rings were observed, which surround the peroxisomal matrix marked by DsRed‐SKL. This pattern is typical for peroxisomal membrane proteins (PMPs) in methanol‐grown *H. polymorpha* cells. As shown in Fig. 2B, Hp32g403‐GFP is not extracted upon carbonate treatment, like the PMP Pex14, indicating that it is an integral membrane protein. As expected, the peroxisomal matrix protein catalase is predominantly observed in the soluble fraction. Western blot analysis of total cell extracts indicated that the levels of Hp32g403‐GFP are similar in glucose and methanol‐grown cells (Fig. 2C).

**Figure 3:**
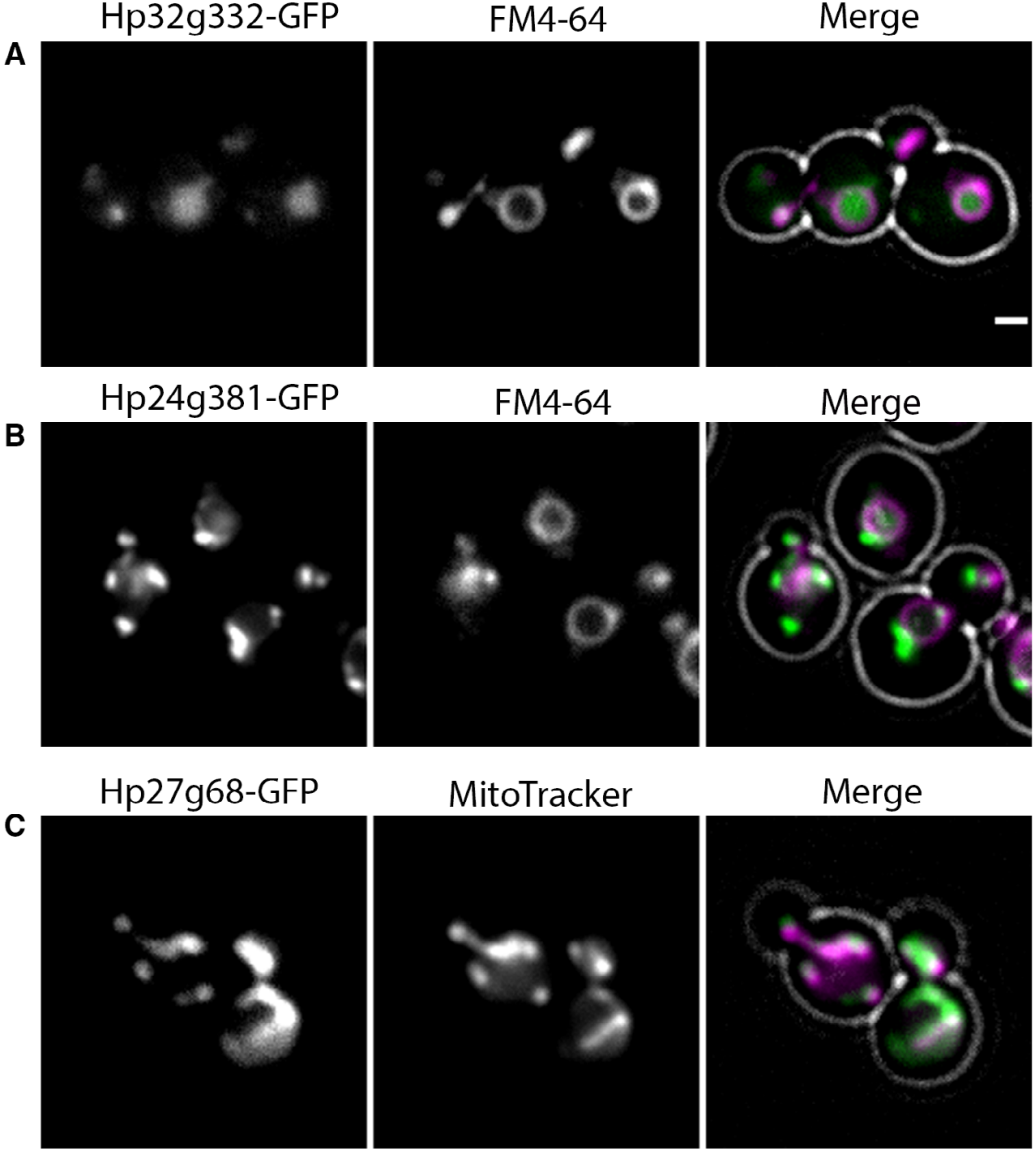
Localization of Hp32g332, Hp24g381, and Hp27g68. FM images of glucose‐/methylamine‐grown WT cells producing P_*AMO*_‐driven (A) Hp32g332‐GFP stained with the vacuole marker FM4‐64, (B) Hp24g381‐GFP stained with the vacuole marker FM4‐64, or (C) Hp27g68‐GFP stained with MitoTracker. Cells were grown to the mid‐exponential growth phase on glucose/methylamine media. In the merged image, the cell contours are indicated in white. Scale bar represents 1 μm. Representative images of two independent experiments are shown.

Cells producing Hp32g332‐GFP, Hp24g381‐GFP, or Hp27g68‐GFP under control of their own promoters displayed very low GFP signals, in both glucose‐ and methanol‐containing media, which severely hampered their localization. We therefore analyzed strains producing these GFP fusion proteins under control of the relatively strong amine oxidase promoter (P_*AMO*_), which is induced by methylamine. In the strain producing Hp32g332‐GFP, GFP fluorescence was predominantly observed in the lumen of the vacuoles (Fig. 3A). Overproduced Hp24g381‐GFP was observed in patch‐like structures at or close to the vacuolar membrane (Fig. 3B). Hp27g68‐GFP localized to discrete network‐like structures that were identified as mitochondria by concurrent staining with the mitochondrion‐specific probe MitoTracker (Fig. 3C), similar as observed for *S. cerevisiae* Sym1 11.

### Hp32g403 is not required for growth on substrates that are metabolized by peroxisomal pathways

**Figure 4:**
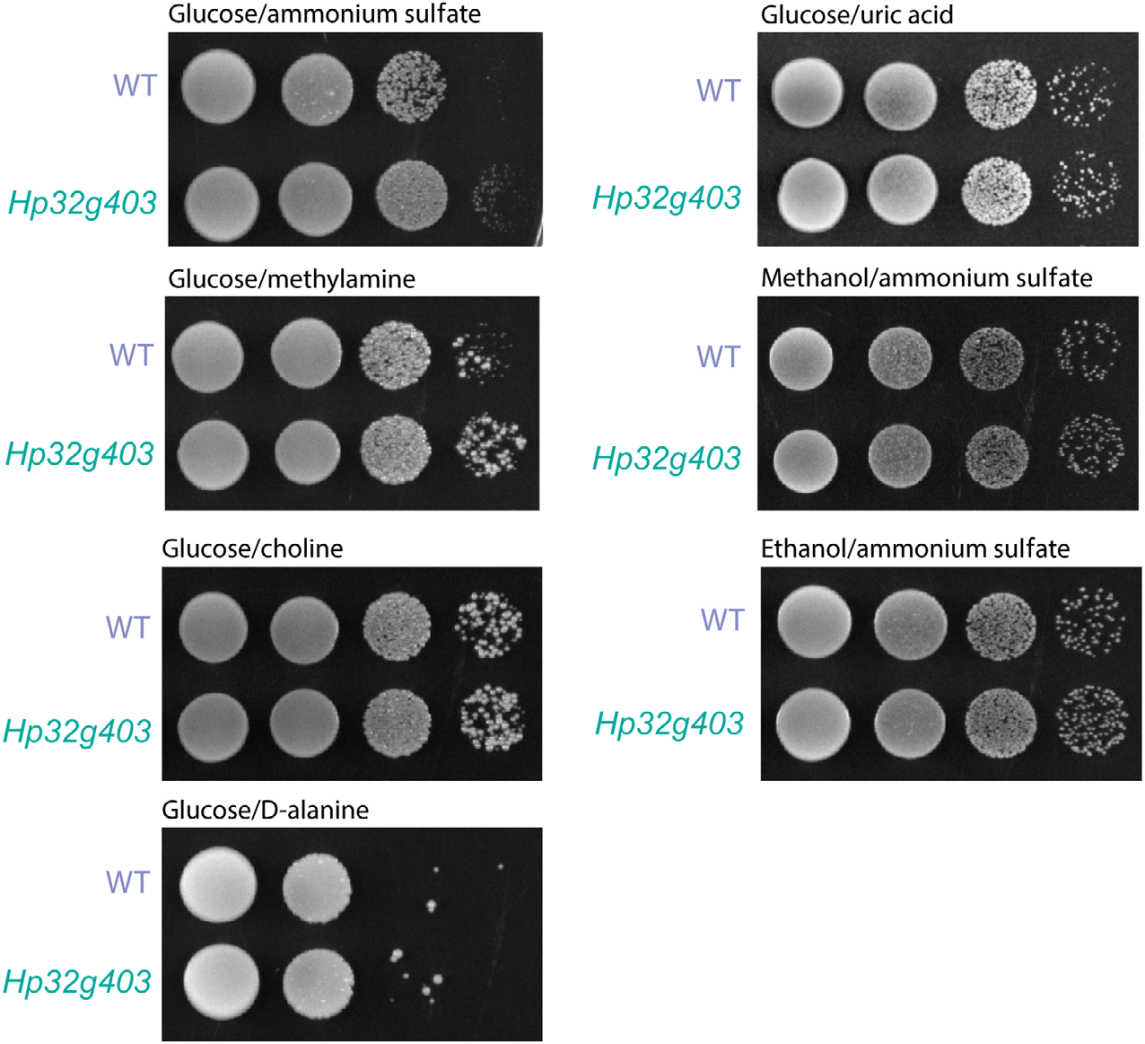
Growth analysis of Hp32g403‐deficient cells. Spot assays performed using WT and Hp32g403‐deficient cells. Cultures were serially diluted and spotted on agar plates containing the indicated carbon and nitrogen sources. A representative spot assay of two independent experiments is shown.

Of all four *H. polymorpha* PXMP2 family proteins tested, only Hp32g403 showed a clear localization to peroxisomes. To analyze a possible pore function of Hp32g403, growth tests were performed using several carbon (methanol, ethanol) and nitrogen sources (methylamine, d‐choline, d‐alanine, uric acid), which are (partially) metabolized by peroxisome borne pathways. Spot tests revealed no significant differences in growth compared to the wild‐type (WT) control for any of the substrates tested (Fig. 4), indicating that Hp32g403 is not an essential, nonspecific pore for transport of metabolites across the peroxisomal membrane at these conditions.

### The absence or overproduction of Hp32g403 affects peroxisome abundance in glucose‐grown cells

**Figure 5:**
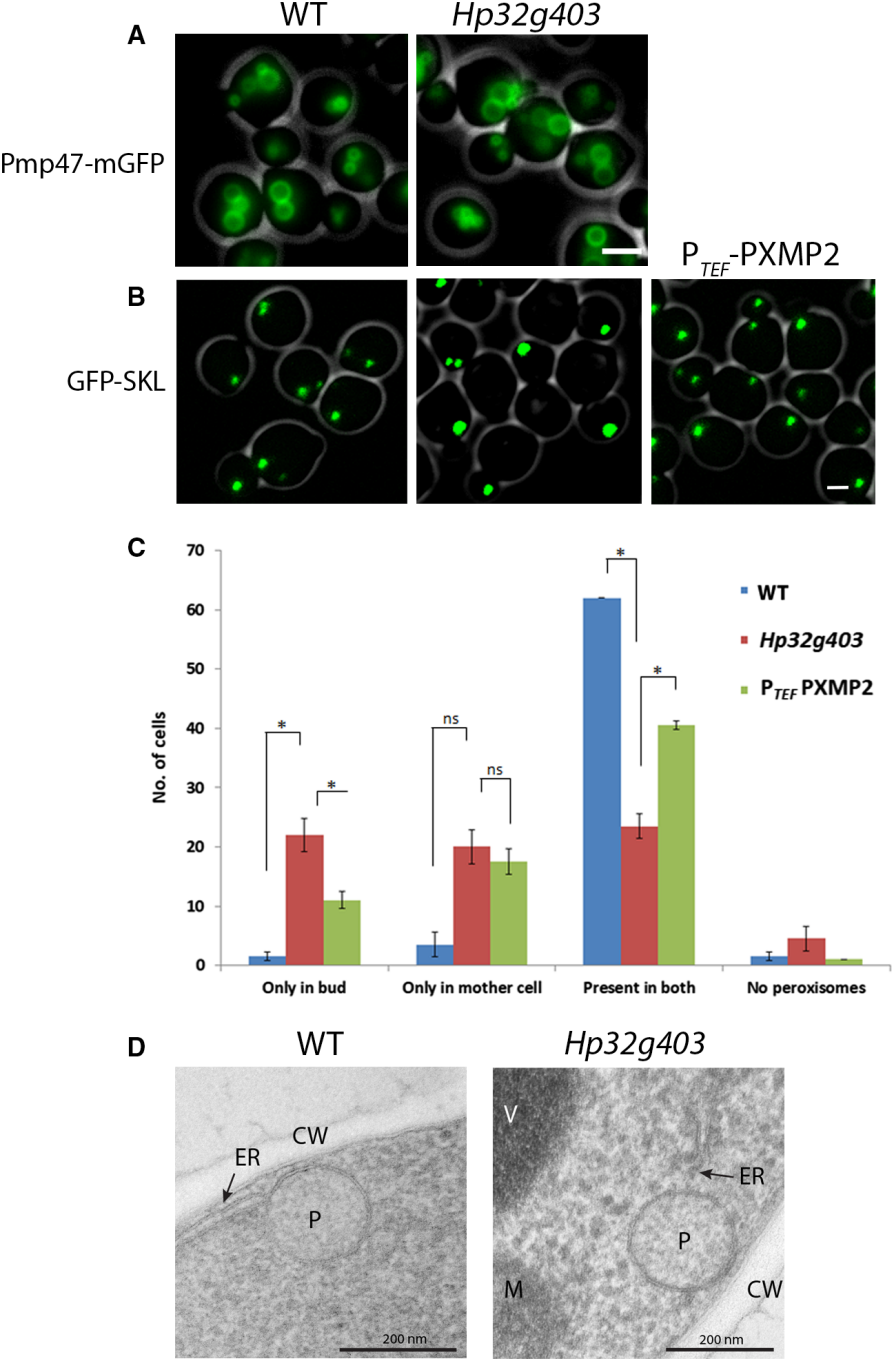
Peroxisome abundance and distribution are altered in glucose‐grown Hp32g403‐deficient cells. (A) CLSM images of methanol‐grown WT and Hp32g403‐deficient cells producing the peroxisomal membrane marker Pmp47‐GFP. Representative images of two independent experiments are shown. (B) CLSM images of WT cells, Hp32g403‐deficient cells, and Hp32g403‐deficient cells expressing P_*TEF*_‐driven human PXMP2. The peroxisomal matrix is marked by GFP‐SKL. Cells were grown to the mid‐exponential growth phase on glucose. The scale bar in A and B represents 1 μm. Representative images of two independent experiments are shown. (C) Organelle quantification (from Z‐stack images) in budding cells of the Hp32g403‐deficient strain with and without P_*TEF*_‐driven human PXMP2, together with the WT control strain, for the presence or absence of peroxisomes in the mother and daughter cells. All strains produced GFP‐SKL as peroxisomal marker. Peroxisomes from 2 × 70 budding cells were counted from two independent experiments. Error bar represents standard deviation. The statistics represent a Student *t*‐test, *P < 0.05. ns—*P* > 0.05. (D) EM analysis of glucose‐grown WT cells and Hp32g403‐deficient cells (CW, cell wall; ER, endoplasmic reticulum; M, mitochondria; P, peroxisome; V, vacuole). Representative cell sections from one experiment are shown.

To investigate whether *H. polymorpha* Hp32g403 plays a role in peroxisome proliferation, we quantified peroxisome numbers in Hp32g403‐deficient cells using confocal laser scanning microscopy (CLSM). This revealed that in methanol‐grown Hp32g403 cells, peroxisome abundance is comparable to that in WT controls (average number of 3.9 ± 0.1 and 3.8 ± 0.2 peroxisomes per cell, respectively; Fig. 5A).

However, the loss of Hp32g403 caused a significant reduction in peroxisome numbers, when cells were grown on glucose (average number of 0.5 ± 0.1 in Hp32g403‐deficient cells relative to 1.0 ± 0.2 in WT controls; Fig. 5B). In glucose cultures of the *H. polymorpha* WT strain, generally a single peroxisome is present in nonbudding cells. This peroxisome divides prior to cell budding and one of the resulting organelles is retained in the mother cell, whereas the other is transported to the bud. Peroxisome quantification confirmed that in budding WT cells, peroxisomes are generally detected in both the mother cell and bud. However, in Hp32g403‐deficient cells, this is only the case in a minor fraction of the cells, whereas substantial percentages of budding cells occur in which peroxisomes are only present in either the mother cell or the bud (Fig. 5C).

In *N. crassa*, WSC plays a role in cortical association of WBs 10. We recently showed that in glucose‐grown *H. polymorpha* WT cells, peroxisomes associate with the plasma membrane and cortical ER 22. Electron microscopy (EM) analysis revealed that in Hp32g403‐deficient cells, peroxisomes remain localized in close vicinity to the plasma membrane and cortical ER (Fig. 5D), suggesting that Hp32g403 is not required for cortical association.

**Figure 6:**
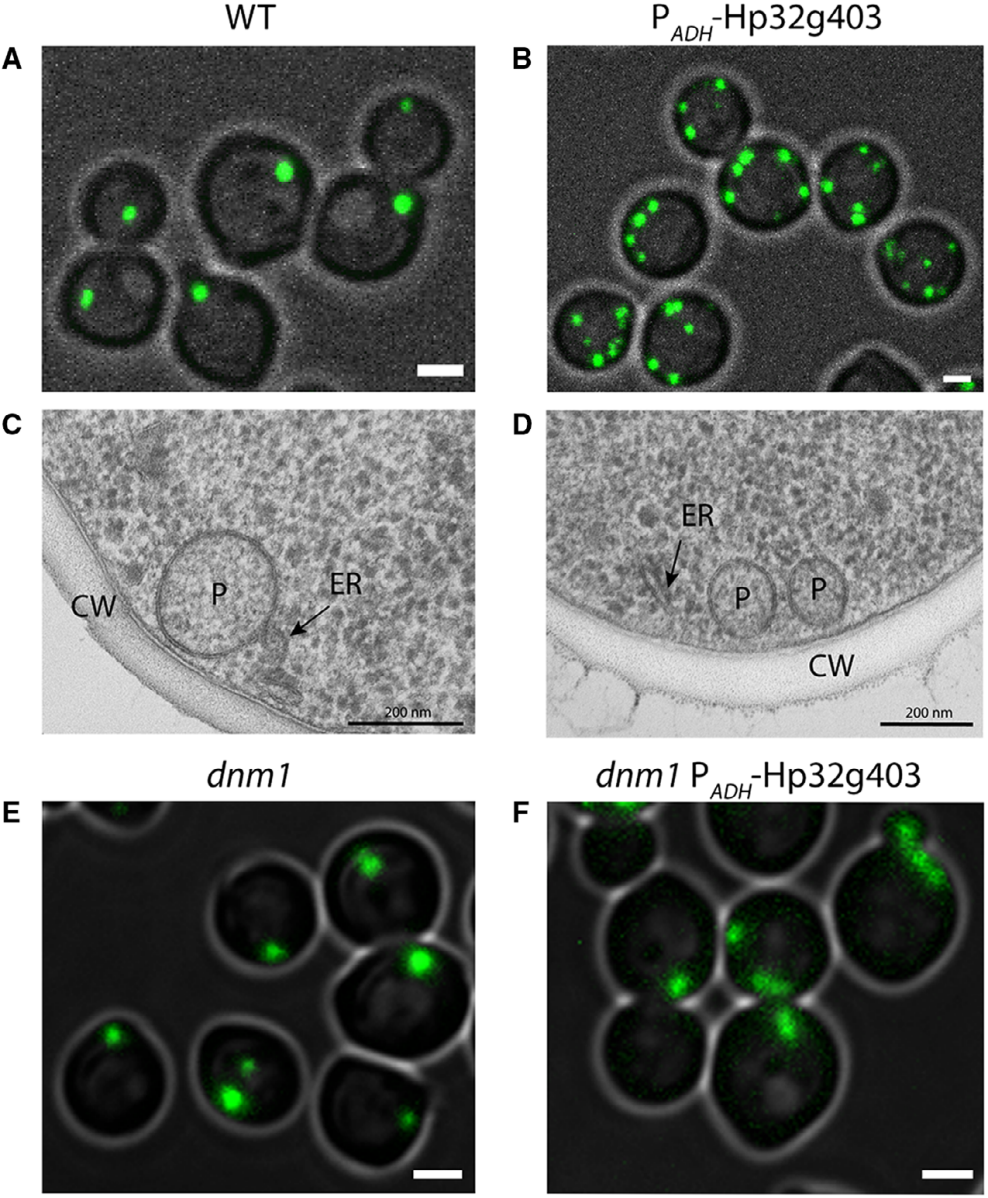
Hp32g403 overproduction results in enhanced numbers of peroxisomes in glucose‐grown cells. FM images of glucose‐grown WT (A) and Hp32g403‐overproducing cells (P_*ADH*_‐Hp32g403) (B). FM images of two independent experiments are shown. EM analysis of WT (C) and the Hp32g403‐overproducing strain (D) (P—peroxisome; CW—cell wall; ER—endoplasmic reticulum). Representative cell sections from one experiment are shown. FM images of glucose‐grown *dnm1* (E) and *dnm1* cells overproducing Hp32g403 (F). Representative images of two independent experiments are shown. In A, B, E, and F, peroxisomes are marked by the matrix protein GFP‐SKL. Scale bars represent 1 μm in A, B, E, and F and 200 nm in C and D.

**Figure 7:**
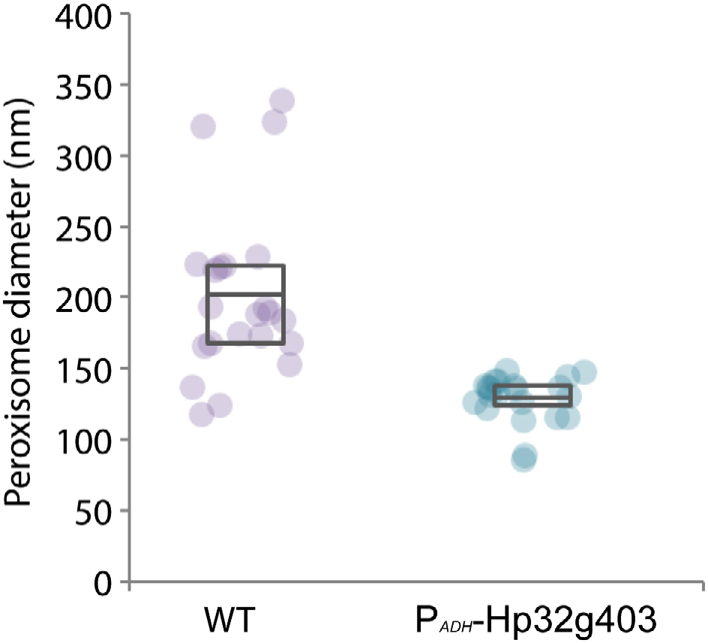
Hp32g403 overproduction results in smaller peroxisomes. Quantification of the peroxisome diameter of glucose‐grown WT and Hp32g403‐overproducing cells using EM. For each strain, one culture was grown and analyzed by EM. For each strain, 22 peroxisomes are measured and depicted in an interquartile box together with the diameter of the individual peroxisomes.

Finally, we analyzed the effect of Hp32g403 overproduction by placing the encoding gene under control of the strong *ADH1* promoter (P_*ADH*_). FM analysis revealed that overproduction of Hp32g403 leads to an increase in GFP‐SKL‐positive fluorescent puncta in glucose‐grown cells (Fig. 6A,B) from 1.17 ± 0.01 peroxisomes per cell in WT controls to 3.06 ± 0.01 in the *PEX37* overexpression strain. In cells of the Pex37 overproduction strain, peroxisome size decreased as evident from EM analysis (Fig. 6C,D; Fig. 7). The peroxisomes invariably were present close to the cell cortex and plasma membrane as evident from FM (Fig. 6A,B) and EM analysis (Fig. 6C,D).

No increase in peroxisome numbers was observed upon overproduction of Hp32g403 in cells lacking the dynamin‐related protein Dnm1, indicating that enhanced levels of Hp32g403 stimulate Dnm1‐dependent peroxisome fission (Fig. 6E,F). Interestingly, peroxisomes are more elongated in *dnm1* cells overproducing Hp32g403 (Fig. 6F) relative to the organelles in *dnm1* control cells (Fig. 6E). Overproduction of Hp32g403 did not affect growth. The optical densities of glucose cultures at the stationary phase (8 h after inoculation) were 3.2 ± 0.0 (WT) and 3.3 ± 0.0 (P_*ADH*_‐Hp32g403) and for methanol cultures (24 h after inoculation) 3.2 ± 0.1 and 3.1 ± 0.2, respectively.

### Human PXMP2 partially rescues the phenotype of Hp32g403‐deficient cells

The human PMP PXMP2 shows 25% amino acid sequence identity with Hp32g403. To investigate whether human PXMP2 is a functional ortholog of Hp32g403, the PXMP2 coding region was expressed in Hp32g403‐deficient cells under control of the P_*TEF*_ promoter. A significant increase in number of cells in which peroxisomes were present in both the mother cell and bud was observed, together with a strong decrease in the number of cells with a peroxisome present only in the bud (Fig. 5C). In addition, the average number of peroxisomes per cell in glucose‐grown cells increased twofold and reached the same value as observed in the WT control (1.0 ± 0.29 and 1.0 ± 0.01, respectively).

**Figure 8:**
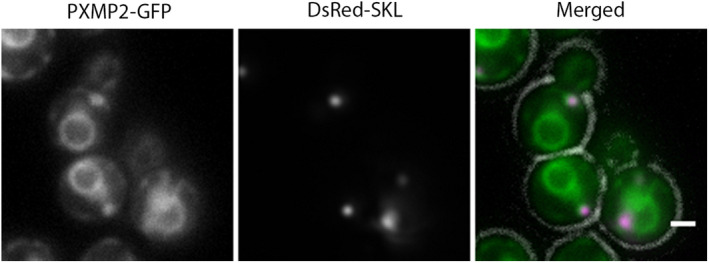
Human PXMP2 partially localizes to peroxisomes in *Hansenula polymorpha*. FM images of Hp32g403‐deficient cells producing PXMP2‐GFP under control of the *TEF* promoter together with P_*ADH*__*1*_‐driven DsRed‐SKL as a peroxisome matrix marker. Cells were grown on glucose medium. Scale bar represents 1 μm. A representative image of two independent cultures is shown.

FM analysis of a strain producing a C‐terminal GFP fusion of PXMP2 under control of the constitutive *TEF* promoter (P_*TEF*_) showed that a portion of protein colocalized with DsRed‐SKL, but most GFP fluorescence was detected at another structure, which, based on its morphology, most likely represents the nuclear envelope (Fig. 8).

## Discussion

Here, we identified *H. polymorpha* Hp32g403, a PXMP2 family protein, which localizes to peroxisomes. Based on sequence analysis, homology to several known membrane proteins and the outcome of our carbonate extraction experiment (Fig. 2B), Hp32g403 most likely is an integral PMP. Our data indicate that this novel yeast protein is required for proper peroxisome multiplication and segregation in cells grown at peroxisome‐repressing growth conditions (glucose), but not at peroxisome‐inducing growth conditions (methanol). Because of its role in regulating peroxisome abundance, we consider this PMP being a peroxin and designated it Pex37.

*H.polymorpha* Pex37 is the third peroxisomal PXMP2 family member that has been identified, in addition to *N. crassa* WSC and mammalian PXMP2. *Neurospora crassa* WSC has been implicated in the formation of WB from peroxisomes and in the inheritance of WBs via cortical association 10. PXMP2 has been proposed to function as nonselective pore in the peroxisomal membrane of mammalian cells. Our data indicate that *H. polymorpha* Pex37 is important for peroxisome multiplication and segregation at peroxisome‐repressing conditions, which is reminiscent of the functions proposed for *N. crassa* WSC.

In glucose‐grown *H. polymorpha pex37* cells, peroxisome multiplication and segregation is abnormal. In glucose‐grown WT cells, the single peroxisome that is present in mother cells divides prior to cell budding. One of the resulting organelles remains in the mother, anchored to the cell cortex by the retention factor Inp1 23, 24. The other organelle is transported to the bud, a process that requires the actin cytoskeleton, the motor protein Myo2, and the inheritance protein Inp2 25, 26. Our data revealed that in glucose‐grown *pex37* cells, peroxisomes do not multiply prior to yeast budding. The single peroxisome either remains in the mother cell or is transported to the bud.

Peroxisome fission can be divided into three steps. First, the organelle elongates, followed by constriction and ultimately the actual scission process. In *H. polymorpha,* Pex11 and Dnm1 are key players in peroxisome fission, in both glucose‐ and methanol‐grown cells 27, 28. In glucose‐grown *H. polymorpha dnm1* cells, the single peroxisome present in the mother cell forms a protrusion into the developing bud and ultimately divides in two organelles during cytokinesis 27. In glucose‐grown *pex11* cells, the organelle does not elongate and the single peroxisome is invariably transported to the bud, leaving the mother cell without a peroxisome 28. Apparently, at these conditions the pulling force of Myo2 toward the bud is stronger than the capacity of Inp1 to retain the single organelle in the mother cell. In *pex37* cells, the peroxisome does not elongate nor divide. In this mutant, the single peroxisome either remains in the mother or moves to the bud, suggesting that the retention force and the pulling force might be similar.

The observation that, like in WT cells, peroxisomes are still localized to the cell periphery in *pex37* cells indicates that Pex37 is not essential for associating peroxisomes to the cell cortex. Instead, our results suggest that in addition to Pex11 and Dnm1, Pex37 is essential for peroxisome fission in *H. polymorpha* cells grown at peroxisome‐repressing conditions. Indeed, like overproduction of *H. polymorpha* Pex11 and Dnm1 27, 28, also Pex37 overproduction results in enhanced peroxisome numbers. Overexpression of *PEX37* in *dnm1* cells does not cause an increase in peroxisome abundance, indicating that the increase in organelle numbers in Pex37‐overproducing cells is due to Dnm1‐dependent peroxisome fission. However, different from Pex11 and Dnm1, Pex37 is not essential for peroxisome multiplication when cells are grown on methanol.

Using *N. crassa* WSC as a query, only PXMP2 is found in *H. sapiens*. But using Hp32g403, no human homologues are found using a variety of tools (HMMER3, HHpred, HHblits, Genome3D, BLASTP). However, we could establish a conservation of function between *H. polymorpha* Pex37 and human PXMP2 through the partial complementation of the *pex37* phenotype by human PXMP2. When *H. polymorpha pex37* cells producing human PXMP2 were grown on glucose, the average number of peroxisomes per cell increased again to similar numbers as observed in WT controls. The peroxisome segregation defect was only partially restored upon introduction of Pxmp1 in *pex37* cells. Possibly, this is related to the fact that the molecular mechanisms of peroxisome segregation are different in human cells.

Mammalian PXMP2 functions as a nonselective pore for solute transports in the peroxisome membrane. This pore allows diffusion of molecules with molecular masses of up to 300 Da 5. We showed that deletion of the *PEX37* gene does not affect growth of *H. polymorpha* on methanol‐ or ethanol‐containing media. Also, the metabolism of D‐amino acids, D‐choline, or methylamine by peroxisomal oxidases was not defective in the *PEX37* deletion strain, indicating that Pex37 is not essential for diffusion of these metabolites into peroxisomes. Methanol metabolism requires import of xylulose 5‐phosphate (230 Da) into peroxisomes, which apparently also does not require Pex37. Interestingly, a recent study in *S. cerevisiae* demonstrated that Pex11 forms a nonselective channel for the transfer of metabolites with size exclusion limit of 300–400 Da across the peroxisomal membrane 6. Hence, it is possible that Pex11 and Pex37 play redundant roles in metabolite transport, explaining why we did not observed growth defects for the *pex37* mutant strain.

*In silico* analysis indicated differences in the number of PXMP2‐related proteins in various species. Sym1 and YOR292c are the sole *S. cerevisiae* PXMP2 proteins, while all other organisms analyzed contained more than two PXMP2 proteins (Table 1). A possible explanation is that *S. cerevisiae* has evolved from an ancestor yeast species that underwent whole‐genome duplication followed by massive gene loss 29.

*H. polymorpha* Hp27g68 showed a mitochondrial localization, like *S. cerevisiae* Sym1 11 and mammalian MPV17 12, 30, 31. *H. polymorpha* Hp24g381 accumulated in patches close to the vacuolar membrane. It is unclear what these patches represent. Because this GFP fusion protein could only be detected upon overproduction, this result should be interpreted with caution. Using the endogenous promoter, the levels of the Hp32g332‐GFP fusion protein were below the limit of detection as well. Upon overproduction, weak fluorescence was predominantly detected in the vacuole lumen. Because Hp32g332 is most likely a membrane protein, Hp32g332‐GFP is probably degraded by autophagy, which could have been stimulated by its overproduction.

Summarizing, PXMP2 proteins are ubiquitously present in eukaryotes. These proteins localize to different intracellular compartments including mitochondria and peroxisomes. In addition to the well‐characterized peroxisome‐localized proteins in fungi (WSC) and mammals (PXMP2), we here show that yeast peroxisomes also harbor a PXMP2 protein, which we call Pex37. Our data indicate that this novel peroxin most likely is involved in peroxisome fission at peroxisome‐repressing growth conditions.

## Materials and methods

### Strains and growth conditions

The *H. polymorpha* strains used in this study are listed in Table 2. Yeast cells were grown at 37 °C in batch cultures on mineral medium (MM) 32 supplemented with 0.5% glucose or 0.5% methanol as carbon sources and 0.25% ammonium sulfate or 0.25% methylamine as nitrogen sources. When required, media were supplemented with amino acids to a final concentration of 30 μg·mL^−1^. For the selection of transformants, YPD plates contained 100 μg·mL^−1^ nourseothricin (Werner Bioagents, Jena, Germany), 100 μg·mL^−1^ zeocin (Invitrogen, Groningen, The Netherlands), or 300 μg·mL^−1^ hygromycin (Invitrogen). For cloning purposes*, Escherichia coli* DH5α was used as host for propagation of plasmids using Luria Broth supplemented with the appropriate antibiotics (100 μg·mL^−1^).

**Table 2:** Yeast strains used this study.

Strains	Characteristics	Reference
WT	NCYC495 *leu1.1*	49
WT. DsRed‐SKL	WT cells with integration of plasmid pHIPX7‐DsRed‐SKL	This study
WT. DsRed‐SKL.Pex37‐mGFP	WT.DsRed‐SKL with integration of plasmid pSEM060	This study
WT. DsRed‐SKL pHIPZ‐Hp32g332‐mGFP	WT.DsRed‐SKL integrated with plasmid pHIPZ‐Hp32g332‐mGFP	This study
WT. DsRed‐SKL pHIPZ‐Hp27g68‐mGFP	WT.DsRed‐SKL integrated with plasmid pHIPZ‐Hp27g68‐mGFP	This study
WT. DsRed‐SKL pHIPZ‐Hp24g381‐mGFP	WT.DsRed‐SKL integrated with plasmid pHIPZ‐Hp27g68‐mGFP	This study
WT.Pex14mKATE2 pHIPZ5‐Hp27g68‐mGFP	WT.Pex14mKATE2 with integrated pHIPZ5‐Hp27g68‐mGFP	This study
WT.Pex14mKATE2pHIPZ5‐Hp24g381‐mGFP	WT.Pex14mKATE2 integrated with pHIPZ5‐Hp24g381‐mGFP	This study
WT.Pex14mKATE2pHIPZ5‐Hp32g332‐mGFP	WT.Pex14mKATE2 integrated with pHIPZ5‐Hp32g332‐mGFP	This study
WT.Pmp47‐GFP	WT cells integrated with plasmid containing P_*PMP47*_ *Pmp47*‐GFP	34
*pex37*. Pmp47‐GFP	*PEX37* deletion strain integrated with plasmid containing P_*PMP47*_ *Pmp47*‐GFP	This study
WT.GFP‐SKL	WT cells integrated with plasmid containing pHIPX7‐GFP‐SKL	28
*pex37*.GFP‐SKL	*PEX37* deletion integrated with plasmid pHIPX7‐GFP‐SKL and P_*PMP47*_ *Pmp47*‐GFP	This study
*pex37*.GFP‐SKL. P_*ADH1*_ *PEX37*	*PEX37* deletion integrated with plasmid pHIPX7‐GFP‐SKL and P_*ADH1*_ *PEX37* plasmid	This study
WT.Pex14mKATE2	WT cells integrated with plasmid containing pHIPH‐Pex14‐mKATE2	This study
*pex37*.P_*ADH1*_ *GFP‐SKL*	*PEX37* deletion strain integrated with plasmid pHIPN18‐GFP‐SKL	This study
*pex37*.P_*ADH1*_ *DsRed‐SKL*	*PEX37* deletion strain integrated with plasmid pHIPN18‐DsRed‐SKL	This study
*pex37*.pHIPZ7‐PXMP2‐2HA. pHIPN18‐eGFP‐SKL	*PEX37* deletion strain integrated with human PXMP2 under P_*TEF*_ and the plasmid pHIPN18‐eGFP‐SKL	This study
*pex37*.pHIPZ7‐PXMP2‐mGFP. pHIPN18‐DsRed‐SKL	*PEX37* deletion strain integrated with human PXMP2‐mGFP under P_*TEF*_ and the plasmid pHIPN18‐DsRed‐SKL	This study
*dnm1*	*DNM1* deletion strain	34
*dnm1*.GFP‐SKL	*DNM1* deletion strain integrated with plasmid pHIPZ7‐GFP‐SKL	This study
*dnm1*.GFP‐SKL P_ADH1_ *PEX37*	*DNM1* deletion strain integrated with plasmid pHIPZ7‐GFP‐SKL and P_*ADH1*_ *PEX37* plasmid	This study

For spot assays, exponential glucose‐growing *H. polymorpha* cells were harvested by centrifugation and diluted to an OD_660_ of 1.0 in H_2_O. Cells were serial diluted (10^−1^, 10^−2^, 10^−3^, 10^−4^, 10^−5^) and spotted on MM plates containing different carbon sources (0.5% glucose, 0.5% methanol or 0.5% ethanol) and nitrogen sources (0.25% ammonium sulfate, 0.25% methylamine, 0.25% choline, 0.25% d‐alanine or 0.25% uric acid). Growth differences were followed during 48 h of incubation at 37 °C.

### Construction of yeast strains

Plasmids and primers used in this study are listed in Tables 3 and 4. Transformation was performed as described previously 33.

**Table 3:** Plasmids used in this study.

Plasmid	Description	Reference
pHIPX7‐DsRed‐SKL	Plasmid containing P_*TEF*_ *‐*DsRed‐SKL, amp^R^, Leu^R^	28
pHIPZ‐mGFP fusinator	pHIPZ plasmid containing mGFP and *AMO* terminator, amp^R^, zeo^R^	26
pSEM060	Plasmid containing C‐terminal part of *PEX37* fused to GFP, amp^R^, zeo^R^	This study
pHIPZ‐Hp32g332‐mGFP	Plasmid containing Hp32g332 fused with GFP, amp^R^, zeo^R^	This study
pHIPZ‐Hp27g68‐mGFP	Plasmid containing Hp27g68 fused with GFP, amp^R^, zeo^R^	This study
pHIPZ‐Hp24g381‐mGFP	Plasmid containing Hp27g68 fused with GFP, amp^R^, zeo^R^	This study
pDONR‐P4‐P1R	Standard Gateway vector	Invitrogen
pDONR‐P2R‐P3	Standard Gateway vector	Invitrogen
pENTR‐221‐HPH	pENTR‐221 containing hygromycin marker, hph^R^, kan^R^	50
pDEST‐R4‐R3	Standard destination vector	Invitrogen
pENTR‐41‐*PEX37* 5′	pDONOR‐P4‐P1 containing 5′ region of Hp32g403, kan^R^	This study
pENTR‐23‐*PEX37* 3′	pDONOR‐P2R‐P3 containing 3′ region of Hp32g403, kan^R^	This study
pSEM027	pDEST‐R4‐R3 containing *PEX37* deletion cassette, Hph^R^, amp^R^	This study
pHIPZ5 Nia	Plasmid containing multiple cloning site and *AMO* promoter, zeo^R^, amp^R^	51
pHIPX7 GFP‐SKL	Plasmid containing GFP‐SKL under the control of *TEF* promoter, Leu^R^, kan^R^	28
pHIPZ7‐GFP‐SKL	Plasmid containing GFP‐SKL under the control of *TEF* promoter, zeo^R^, amp^R^	52
pHIPZ5‐Hp27g68‐mGFP	Plasmid containing Hp27g68 fused to GFP under control of P_*AMO*_, zeo^R^, amp^R^	This study
pHIPZ5‐Hp24g381‐mGFP	Plasmid containing Hp24g381 fused to GFP under control of P_*AMO*_, zeo^R,^ amp^R^	This study
pHIPZ5‐Hp32g332‐mGFP	Plasmid containing Hp32g332 fused to GFP under control of P_*AMO*_, zeo^R^, amp^R^	This study
pHIPZ‐PMP47‐mGFP	Plasmid containing PMP47‐mGFP under the control of P_*PMP47*_, zeo^R^, amp^R^	53
pHIPZ18‐eGFP‐SKL	pHIPZ containing eGFP.SKL under control of P_*ADH1*_, zeo^R^, amp^R^	This study
pHIPZ4‐GFP‐SKL	pHIPZ4 containing eGFP.SKL, zeo^R^, amp^R^	54
pHIPN18‐eGFP‐SKL	pHIPN containing eGFP.SKL under control of P_*ADH1*_, nat^R^, amp^R^	This study
pHIPN4	Plasmid containing amp^R^, nat^R^	53
pHIPN18‐*PEX37*	pHIPN containing *PEX37* under control of P_*ADH1*_, nat^R^, amp^R^	This study
pHIPN18‐DsRed‐SKL	pHIPN containing DsRed.SKL under control of P_*ADH1*_, nat^R^, amp^R^	This study
pHIPZ4‐DsRed‐SKL	Plasmid containing DsRed.SKL, zeo^R^	55
pHIPZ7‐PXMP2‐2HA	pHIPZ containing human *PXMP2* fused with 2HA tag under control of P_*TEF*_, zeo^R^, amp^R^	This study
pUC57‐PXMP2 plasmid	pUC57 containing human *PXMP2* fused with 2HA tag	This study
pHIPZ7	pHIPZ plasmid containing *TEF1* promoter, zeo^R^, amp^R^	56
pHIPZ7‐PXMP2‐mGFP	pHIPZ containing human *PXMP2* fused with GFP under control of P_*TEF*_, zeo^R^, amp^R^	This study

**Table 4:** Primers used in this study.

P1	5′ AAAAAGCTTATGCTCGCCGATCTGAAC 3′
P2	5′ TTTAGATCTTTCATTCTTGTTCTGTTC 3′
Hp32g332 Fwd	5′ AAAAAGCTTACTGGCAGCTTCTGA 3′
Hp32g332 Rev	5′ AAGGATCCCGTGATCAGAGTCAGTAG 3′
P3	5′AAAAAGCTTATGATCACTGGATACAAAACGCTC3′
P4	5′ AAAAGATCTCTGTCCACTGTGCTCAACC 3′
P5	5′ GCTCTCATGCCTATCAG 3′
P6	5′AAAGGATCCGCTGGTAGCATTCCTCAAG 3′
Fwd attB4	5′GGGGACAACTTTGTATAGAAAAGTTGCCGCTCCGCCTCTTGGTGCTCCTCTAA3′
Rev attb1	5′GGGGACTGCTTTTTTGTACAAACTTGGCAAAGGGACGCGTTTTGTGACAGAG3′
Fwd attB2	5′GGGGACAGCTTTCTTGTACAAAGTGGCCACCAGTGGGCCGTGTTCTTC3′
Rev attB3	5′GGGGACAACTTTGTATAATAAAGTTGCGTGGACAAGGGCCGTCATAAACTGT3′
PEX37 del. Fwd	5′GCTCCGCCTCTTGGTGCTCCTCTAA3′
PEX37 del. Rev	5′GTGGACAAGGGCCGTCATAAACTGT3′
P7 (Hp27gBamHI‐F)	5′CGGGATCCATGAGAGCAGTTATCTACGGAGG3′
P8 (Hp27gNdeI‐R)	5′CCCATATGGGATCTGAACCTCGACTTTCTG3′
P9 (Hp24gBamHI‐F)	5′CGGGATCCATGTCACGTGTTATTTCTTTTTCTAG3′
P10 (Hp24gNdeI‐R)	5′CCCATATGGGATCTGAACCTCGACTTTCTG3′
P11 (Hp32gBamHI‐F)	5′CGGGATCCATGCCCGAAGAAGTGCTG3′
P12 (Hp32gSpeI‐R)	5′CCACTAGTGGATCTGAACCTCGACTTTCTG3′
Adh1‐F	5′AAGGAAAAAAGCGGCCGCCCCCTGCATTATTAATCACC3′
Adh1‐R	5′AATCAATCAATCAATTTAAAAAGCTTGGG3′
PEX37 fw	5′CCCAAGCTTATGCTCGCCGATCTGAACAG3′
PEX37 rev	5′TCTAGAGGAGGCATTGTGGACA3′
PTEFNruI_F	5′CCCTCGCGACATGGAACCAAGACCCATGAC3′
TEFPxmp2BglII_R	5′GAAGATCTTTTACCCAAAGAAGCCAAATAAG3′

### Plasmid constructions

Plasmid pSEM060 was constructed by PCR amplification of Hp32g403 gene lacking the stop codon using the primers P1 and P2 and *H. polymorpha* genomic DNA as a template. The obtained PCR fragment was digested with HindIII and BglII and inserted between the HindIII and BglII sites of the pHIPZ‐mGFP fusinator plasmid. The resulting plasmid containing a *PEX37*‐mGFP fusion gene, designated as pSEM060, was linearized with PflMI and integrated into the *PEX37* gene of *H. polymorpha* WT strain producing DsRed‐SKL.

Similarly, plasmid pHIPZ‐Hp32g332‐mGFP (C‐terminal fusion) was constructed by PCR amplification of the Hp32g332 gene without a stop codon, using primers Hp32g332 Fwd and Hp32g332 Rev and *H. polymorpha* genomic DNA as a template. The obtained DNA fragment was digested with HindIII and BamHI and cloned into the HindIII–BglII‐digested pHIPZ‐mGFP fusinator plasmid. The resulting plasmid was linearized with PflMI and integrated into the Hp32g332 gene of *H. polymorpha* WT producing DsRed‐SKL as a peroxisomal matrix marker.

Plasmid pHIPZ‐Hp27g68‐mGFP was constructed by PCR amplification of the Hp27g68 gene lacking a stop codon using the primers P3 and P4 and *H. polymorpha* genomic DNA as a template. The obtained PCR product was digested with HindIII and BglII and inserted between the HindIII and BglII sites of the pHIPZ‐mGFP fusinator plasmid. The resulting plasmid encoding a Hp27g68‐mGFP fusion protein was linearized with BsmI and integrated into Hp27g68 gene of *H. polymorpha* WT strain producing DsRed‐SKL.

Plasmid pHIPZ‐Hp24g381‐mGFP was constructed by PCR amplification of the Hp24g381 gene lacking a stop codon using the primers P5 and P6. pHIPZ‐mGFP fusinator was linearized with HindIII, treated with Klenow fragment followed by digestion with BglII. The linearized plasmid was ligated to the BamHI‐digested PCR fragment. The resulting plasmid was linearized with BglII and integrated in *H. polymorpha* WT producing DsRed‐SKL.

The plasmids pHIPZ5‐Hp27g68‐mGFP, pHIPZ5‐Hp24g381‐mGFP, and pHIPZ5‐Hp32g332‐mGFP were constructed by PCR amplification of the respective genes with the GFP tag lacking the stop codon by using genomic DNA of *H. polymorpha*, containing endogenous Hp27g68‐GFP, Hp24g381‐GFP, and Hp32g332‐GFP fusion constructs, as a template and primer combinations P7 + P8, P9 + P10, and P11 + P12, respectively. The amplified DNA Hp27g68‐GFP and Hp24g38‐GFP was digested using BamHI and NdeI, whereas Hp32g332‐GFP was digested using BamHI and SpeI. The plasmid pHIPZ5‐Nia was also digested with the same restriction enzyme combinations for the particular gene. The amplified and digested gene fragments were ligated to the respective plasmid fragment. The resulting plasmids expressing a fusion gene were linearized using Bsu36I and transformed into *H. polymorpha* WT strain containing Pex14‐mKATE2.

Plasmid pHIPZ18‐eGFP‐SKL was constructed by performing PCR using primers Adh1‐F and Adh1‐R on *H. polymorpha* genomic DNA, followed by digestion of the PCR product with HindIII and NotI. The resulting fragment was inserted between the HindIII and NotI sites of pHIPZ4‐GFP‐SKL.

For the construction of pHIPN18‐eGFP‐SKL, digestion of plasmids pHIPZ18‐eGFP‐SKL and pHIPN4 was performed with NotI and XhoI, followed by ligation and transformation into *E. coli*. Plasmid pHIPN18‐PEX37 was constructed by amplification of the *PEX37* ORF plus terminator region (975 bp) with additional HindIII and XbaI sites in a PCR using primers PEX37 fw and PEX37 rev and *H. polymorpha* genomic DNA as a template, followed by digestion of the PCR product with HindIII and XbaI. The PCR fragment was inserted between the HindIII and XbaI sites of pHIPN18‐eGFP‐SKL. The resulting plasmid was linearized with PstI and integrated in the *H. polymorpha pex37* strain containing pHIPX7‐GFP‐SKL plasmid.

pHIPN18‐DsRed‐SKL was created using plasmids pHIPZ4‐DsRed‐SKL and pHIPN18‐eGFP‐SKL. Both plasmids were digested using HindIII and SalI, followed by ligation.

Plasmid pHIPZ7‐GFP‐SKL was linearized with MunI and integrated in *dnm1* 34 cells. Subsequently, plasmid pHIPN18‐Pex37 was linearized with PstI and integrated in this strain.

### Construction of a plasmid containing human PXMP2

The human PXMP2 cDNA was codon‐optimized for expression in *Pichia pastoris* by OptimumGene™ algorithm (GenScript HK Limited, Hongkong, China). Codon‐optimized PXMP2 containing two human influenza hemagglutinin (HA) tags was subcloned in pUC57 vector (GenScript HK Limited). Plasmid pHIPZ7‐PXMP2‐2HA was constructed by digesting pUC57 containing PXMP2 and pHIPZ7 using restriction enzymes HindIII and XbaI, followed by ligation. The resulting plasmid was linearized using MunI and transformed into *H. polymorpha pex37* containing pHIPN18‐eGFP‐SKL.

To construct human PXMP2‐GFP, pHIPZ7‐PXMP2‐2HA was used as a template to amplify P_TEF_‐PXMP2 using primers PTEFNruI_F and TEFPxmp2BglII_R. The pHIPZ‐mGFP fusinator plasmid as well as the amplified PXMP2 fragment was digested using NruI and BglII, followed by ligation. The resulted plasmid, designated P_*TEF*_‐PXMP2‐mGFP, was linearized using MunI and transformed into *H. polymorpha pex37* containing pHIPN18‐DsRed‐SKL.

### Construction of Gateway plasmids

A *H. polymorpha PEX37* (Hp32g403) deletion strain was constructed by replacing the portion of the genomic region of Hp32g403 comprising nucleotides +1659 to +2008 by the antibiotic marker Hygromycin (Hph). To this end, pSEM027 [pDest‐*PEX37* (Hp32g403) deletion cassette)] was constructed using Invitrogen Gateway Technology (Groningen, The Netherlands). By using *H. polymorpha* genomic DNA as a template, two DNA fragments comprising the regions −1231 to +1658 and +2008 to +2408 bp of the *PEX37* genomic region were obtained by PCR using primers Fwd *att*B4/Rev attb1 and Fwd attB2/Rev attB3, respectively. The PCR fragments were recombined into the vectors pDONR‐P4‐P1R and pDONR‐P2R‐P3, respectively, resulting in the entry vectors pENTR‐*PEX37* 5′ and pENTR‐*PEX37* 3′. Recombination of the entry vectors pENTR‐*PEX37* 5′, pENTR‐221‐HPH, and pENTR‐*PEX37* 3′, and the destination vector pDEST‐R4‐R3, resulted in pSEM027. A 2.6‐kb fragment of pSEM027 comprising the *PEX37* deletion cassette was amplified by PCR with the primers *PEX37* del. Fwd and *PEX37* del. Rev. The amplified fragment was transformed into *H. polymorpha* WT cells producing Pmp47‐GFP as a peroxisomal membrane marker. The deletion was confirmed by PCR and Southern blot analysis. The plasmid pHIPX7 GFP‐SKL was linearized with StuI in the *TEF1* region and transformed into *pex37* cells.

### Fluorescence microscopy

Wide‐field images were made using a Zeiss Axioscope fluorescence microscope (Carl Zeiss, Sliedrecht, the Netherlands). Images were taken using a CoolSNAP HQ2 digital camera and micro‐manager 1.4 software (Photometrics CoolSNAP HQ2, Birmingham, UK). The GFP signal was visualized by using a 470/40‐nm band‐pass excitation filter, a 495‐nm dichromatic mirror, and a 525/50‐nm band‐pass emission filter. DsRed, FM4‐64, and MitoTracker fluorescence was visualized with a 546/12‐nm band‐pass excitation filter, a 560‐nm dichromatic mirror, and a 575/640‐nm band‐pass emission filter. The vacuolar membranes were stained with FM4‐64 (Invitrogen) by incubating cells at 37 °C with 2 μm FM4‐64. Mitochondria were stained with 0.5 μg·mL^−1^ MitoTracker orange (Invitrogen) by incubating cells at 37 °C, followed by extensive washing.

Confocal imaging was performed on a Carl Zeiss LSM800 confocal microscope. For quantification of peroxisomes, Z‐stack images of cells were taken using a 100 × 1.40 NA objective and zen 2009 software (Carl Zeiss). GFP signal was visualized by excitation with a 488‐nm argon laser (Lasos, Jena, Germany), and emission was detected using a 500‐ to 550‐nm band‐pass emission filter. The DsRed signal was visualized by excitation with a 543‐nm helium neon laser (Lasos), and emission was detected using a 565‐ to 615‐nm band‐pass emission filter. Image analysis was carried out using imagej (Bethesda, MA, USA) and adobe photoshop CS6 software (San Jose, CA, USA).

To quantify peroxisome inheritance in WT and *pex37* cells, the cells were grown on glucose‐containing media to the mid‐exponential growth stage. Only cells for which the bud volume was < 25% of the mother cell volume were counted. Quantification was performed manually using two independent cell cultures (70 cells per culture). The images were also used for the quantification of average peroxisome numbers (two independent cultures, 100 cells per culture). The peroxisome number per cell was quantified by counting the number of fluorescent spots per cell for both glucose‐ and methanol‐grown cells. For the quantification of peroxisome numbers in the *PEX37*, overexpression strain cells were grown on glucose and Z‐stacks were prepared by CLSM. Fluorescent spots were counted in cells from two independent cultures. A total of 100 cells were quantified per culture.

Statistical differences were determined by using a Student *t*‐test. Error bars represent standard deviations.

### Electron microscopy

*Hansenula polymorpha* cells were cryo‐fixed using self‐pressurized rapid freezing 35. The copper capillaries were sliced open longitudinally and placed on frozen freeze‐substitution medium containing 1% osmium tetroxide, 0.5% uranyl acetate, and 5% water in acetone. The cryo‐fixed cells were dehydrated and fixed using the rapid freeze‐substitution method 36. Samples were embedded in Epon, and ultrathin sections were collected on formvar‐coated and carbon‐evaporated copper grids. For morphological studies, ultrathin sections were inspected using a CM12 (Philips, Eindhoven, The Netherlands) transmission electron microscope.

### Phylogenetic analysis

Homology‐based searches in the *H. polymorpha* genome sequence 37 were performed as described previously 38. Phylogenetic profiling of the PXMP2‐related proteins was based on a multiple sequence alignment created with ClustalOmega 39 with default parameters and manually curated in Jalview 40. The resulting curated MSA was used to create a phylogenetic tree with phyml 3.1 41 using the LG matrix, 100 bootstraps, tree and leaves refinement, SPR moves, and amino acid substitution rates determined empirically. Secondary structure, transmembrane helices, and disorder predictions were realized with PSIPRED 42, TMHMM 43, and IUP software packages 44, respectively, and drawn with Foundation (http://pvcbacteria.org/foundation) 45.

### Biochemical techniques

An organellar fraction (P3) was obtained as described previously 46 and subjected to carbonate extraction for 30 min on ice, followed by centrifugation for 30 min at 100 000 ***g*** at 4 °C 47. Total cell extracts were prepared from cells treated with 12.5% trichloroacetic acid and used for SDS/PAGE as described previously 48. Equal amounts of protein were loaded per lane. Blots were decorated with mouse monoclonal antisera against GFP (sc‐9996; Santa Cruz Biotechnology, Heidelberg, Germany) or specific polyclonal antisera against Pex14, or catalase. Pyruvate carboxylase‐1 was used as a loading control.

## Conflicts of interest

The authors declare no conflict of interest.

## Author contributions

RS, SM, and IJvdK conceived the project and wrote the original draft; and all authors performed the experiments, analyzed the data, prepared the figures, and contributed to reviewing and editing the manuscript.
